# The 'cardiac-lung mass' artifact: an echocardiographic sign of lung atelectasis and/or pleural effusion

**DOI:** 10.1186/cc7021

**Published:** 2008-09-30

**Authors:** Andreas Karabinis, Theodosios Saranteas, Dimitrios Karakitsos, Daniel Lichtenstein, John Poularas, Clifford Yang, Christodoulos Stefanadis

**Affiliations:** 1 Department of Intensive Care Medicine, General Hospital of Athens, Mesogeion Avenue, Athens, 115 27, Greece; 2 Ambroise-Paré Hospital, Faculté Paris-Ouest, Paris, Boulogne, F-92100, France; 3 Department of Diagnostic Imaging and Therapeutics, University of Connecticut Health Center, Farmington, CT 06030, USA; 4 1st Cardiology Department, Athens University Medical School, Hippokration Hospital, V. Sofias, Athens, 115 27, Greece

## Abstract

**Introduction:**

We conducted an ultrasound study to investigate echocardiographic artifacts in mechanically ventilated patients with lung pathology.

**Methods:**

A total of 205 mechanically ventilated patients who exhibited lung atelectasis and/or pleural effusion were included in this 36-month study. The patients underwent lung echography and transthoracic echocardiography, with a linear 5 to 10 MHz and with a 1.5 to 3.6 MHz wide-angle phased-array transducer, respectively. Patients were examined by two experienced observers who were blinded to each other's interpretation.

**Results:**

A total of 124 patients (60,48%) were hospitalized because of multiple trauma; 60 patients (29,26%) because of respiratory insufficiency, and 21 (10,24%) because of recent postoperative surgery. The mean duration ( ± standard deviation) of hospitalization was 35 ± 27 days. An intracardiac artifact was documented in 17 out of 205 patients (8.29%) by echocardiography. It was visible only in the apical views, whereas subsequent transesophageal echocardiography revealed no abnormalities. The artifact consisted of a mobile component that exhibited, on M-mode, a pattern of respiratory variation similar to the lung 'sinusoid sign'. Lung echography revealed lung atelectasis and/or pleural effusion adjacent to the heart, and a similar M-mode pattern was observed. The artifact was recorded within the left cardiac chambers in 11 cases and within the right cardiac chambers in six.

**Conclusions:**

Lung atelectasis and/or pleural effusion may create a mirror image, intracardiac artifact in mechanically ventilated patients. The latter was named the 'cardiac-lung mass' artifact to underline the important diagnostic role of both echocardiography and lung echography in these patients.

**Trial registration:**

This trial is ISRCTN registered: ISRCTN 49216096.

## Introduction

Incidental echocardiographic artifacts may be due to a distortion of an actual structure from deviation of the ultrasound wave [1-3]. Various causes of echocardiographic artifacts have been described, ranging from silicon breast implants to pacemaker leads [4-7]. Sometimes, the cause may not be obvious and thus the generation of artifacts may be attributed to technical issues such as a poor acoustic window and/or the physical nature of the ultrasound beam. Echocardiographic artifacts may lead the clinician to misdiagnosis of features such as thrombus and valve anomalies, including endocarditis and cardiac tumours, especially in the intensive care unit (ICU) setting [8-12].

In recent years, bedside chest sonography has increasingly been used in the management of critically ill patients to optimize diagnostic and therapeutic procedures [13]. Bedside chest sonography includes modalities such as lung echography and echocardiography; both may provide invaluable information to the clinician [14-18]. In this report, we describe how the application of the two modalities has led us to discover an echocardiographic artifact that is generated by pathologic alterations in the lung parenchyma.

## Materials and methods

### Initial observations

During routine transthoracic echocardiography a persistent yet peculiar cardiac 'mass' was documented in five critically ill patients (three male; age range 22 to 65 years). Two patients were hospitalized because of multiple trauma and three because of postsurgical respiratory insufficiency. All patients were intubated and mechanically ventilated (Servo-I ventilator; Maquet Inc., Bridgewater, NJ, USA).

Lung echography, routinely performed in these patients, revealed areas of atelectasis and or/pleural effusion adjacent to the heart. We utilized a Philips XD11 XE ultrasound device (Philips, Andover, MA, USA) equipped with a linear 5 to 10 MHz transducer to perform lung echography. Patients were examined in the supine position, and a systematic protocol of examination was adhered to. First, the operator located the position of the diaphragm. In this way, anomalies of the lung parenchyma, usually in dependant and dorsal lung regions, could be easily distinguished from liver or spleen [18]. Using anterior and posterior-axillary lines as anatomical landmarks, each chest wall was divided into six lung regions: upper and lower parts of the anterior, upper and lower parts of the lateral, and upper and lower parts of the posterior chest wall [19]. Within a given region of interest, all regional lung was scanned via adjacent intercostal spaces with good acoustic windows. The examination for both lungs took about 20 minutes.

Massive lung edema, lobar bronchopneumonia, pulmonary contusion, and lobar atelectasis all exhibit a massive loss of lung aeration that enables ultrasound transmission deep into the thorax. Lung consolidation appears as poorly defined, wedge-shaped, hypoechoic tissue. Hyperechoic punctiform structures can be seen within consolidation, corresponding to air bronchograms (air-filled bronchi surrounded by consolidated lung parenchyma) [19-21]. Pleural effusion may also appear on longitudinal views, next to dependant lung between the chest wall and the diaphragm. If present, pleural effusion – with hypoechoic to anechoic appearance – is observed during all phases of expiration and inspiration [22,23].

All patients underwent routine transthoracic echocardiographic evaluation. Standard M-mode, two-dimensional echocardiography, and Doppler measurements of left ventricular function were conducted with the same ultrasound device (as mentioned above), equipped with a 1.5 to 3.6-MHz wide-angle, phased-array transducer, in accordance with the recommendations of the American Society of Echocardiography [24]. All usual two-dimensional, transthoracic echocardiographic views (apical to parasternal) were observed and images were stored as digital files for offline analysis.

**Figure 1:**
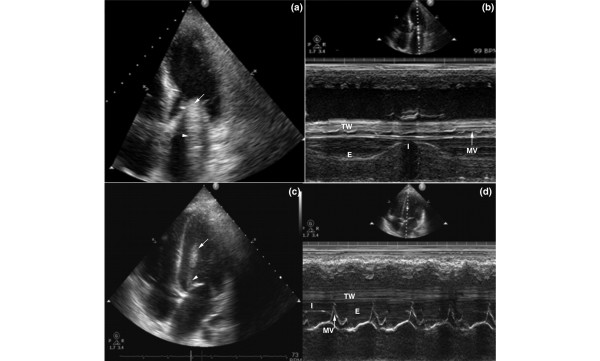
**Echocardiography depicting the artifact (arrows) E, expiration; I, inspiration; MV, mitral valve; TW, thoracic wall**. **(a,c) **Apical four-chamber views and **(b,d) **M-mode of the artifact in the left ventricle. The immobile part (arrow) and the mobile one (arrowhead) may be observed. The respiratory variation that resembles the lung 'sinusoid sign' is either fully (panel b) or partially (panel d) visible.

**Figure 2:**
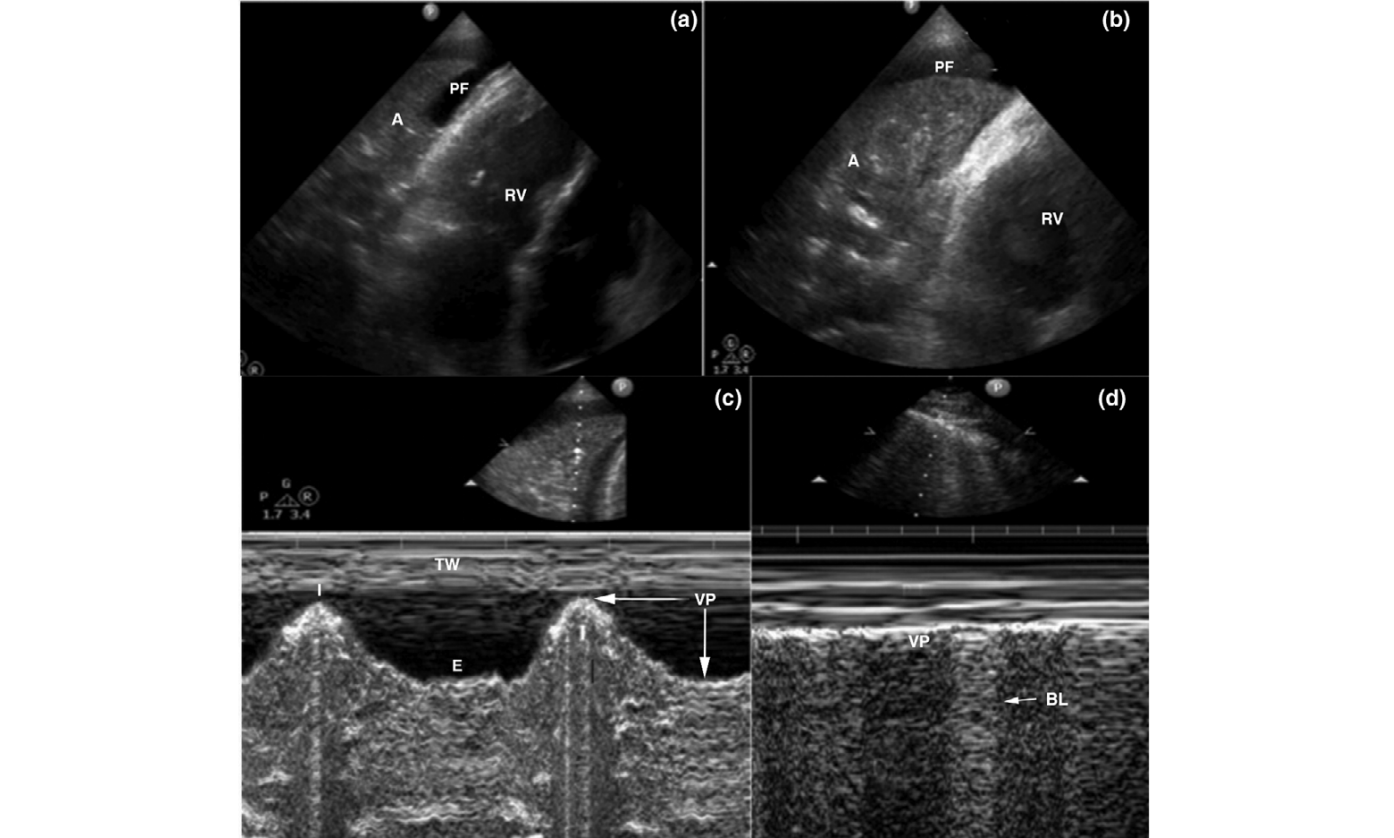
**Lung echography (panel d: normal) (A, atelectasis; BL, b-lines; PF, pleural fluid; RV, right ventricle; VP, visceral pleura)**. **(a,b) **Two-dimensional and (c,d) M-mode lung echography depicting atelectasis and pleural fluid adjacent to the heart. The respiratory variation of the atelectatic lung (panel c) versus the opposite normal lung (panel d) in the same patient may be observed. A, atelectasis; BL, b-lines; E, expiration; I, inspiration; PF, pleural fluid; RV, right ventricle; TW, thoracic wall; VP, visceral pleura.

The echocardiographic artifact was identified in the apical views and exhibited two configurations. In the first configuration (two out of five cases), the 'mass' consisted of two components: an echogenic structure located at the level of the mitral valve, and a less echogenic, mobile structure that projected in a linear manner from the first echogenic structure toward the left atria (Figure 1). However, the 'mass' was not visible in the parasternal views, and color Doppler revealed no abnormalities. Interestingly, the M-mode demonstrated a pattern of respiratory variation resembling the lung 'sinusoid sign' (Figure 1), consistent with pleural effusion [18]. Lung echography performed with 2 to 5 MHz curved transducer, revealed atelectatic areas and pleural effusion adjacent to the heart, furthermore a similar M-mode pattern was documented (Figure 2).

The second configuration of the 'mass' was detected in three out of five patients and had the same basic characteristics, consisting of the same two components as described above (Figure 1). However, the pattern of respiratory variation was not fully apparent, presumably because of the mass being visualized closer to the left ventricular wall. Hence, the motion of the mitral valve and the left ventricle may have overlapped the movement of the 'mass' (Figure 1). Lung echography demonstrated, as in the first case, atelectatic areas and/or pleural fluid adjacent to the heart (Figure 2). The artifact was observed only in the apical views, but it was persistently visible in multiple planes.

Subsequently, transesophageal echocardiography (TEE) was performed in all patients, which failed to reveal the 'mass'. During follow up, three patients died and two patients underwent successful weaning and were finally discharged. In the latter patients, anomalies of the lung parenchyma resolved after extubation, and the above-described corresponding echocardiographic findings also resolved.

### Follow-up study

Patients were recruited from a cohort of 310 critically ill patients who were hospitalized in two units from 2005 until 2008. We performed a 36-month observational study in 205 critically ill patients (120 males and 85 females; age, body mass index and Acute Physiology and Chronic Health Evaluation [APACHE] II score [all mean ± standard deviation]: 45.4 ± 16.9 years, 22.9 ± 5.9 kg/m^2^, and 19 ± 5.2, respectively) who exhibited the same initial findings on lung echography, namely lung atelectasis and/or pleural effusion, in order to investigate the occurrence of similar echocardiographic artifacts. A total of 124 patients (60.48%) were hospitalized because of multiple trauma, 60 patients (29.26%) because of respiratory insufficiency, and 21 (10.24%) because of recent surgery. The mean ( ± standard deviation) length of stay in the ICU was 35 ± 27 days. All patients were intubated and mechanically ventilated during the study period (Servo-I ventilator; Maquet Inc.).

For all patients, family members provided written, informed consent. The study was conducted in accordance with the principles outlined in the Declaration of Helsinki and was approved by the Institutional Ethics Committee. All patients were scanned by two independent experienced observers who were blinded to each others' interpretation. Each observer performed at least 20 two-dimensional scanning sequences of general chest ultrasound daily in each individual patient. Ultrasound examinations were performed throughout each patient's stay in the ICU. All images were stored as digital files and analyzed offline (QLAB, Philips, Bothell, WA, USA). We utilized the same echocardiographic and lung echographic protocols, as described above.

## Results

**Figure 3:**
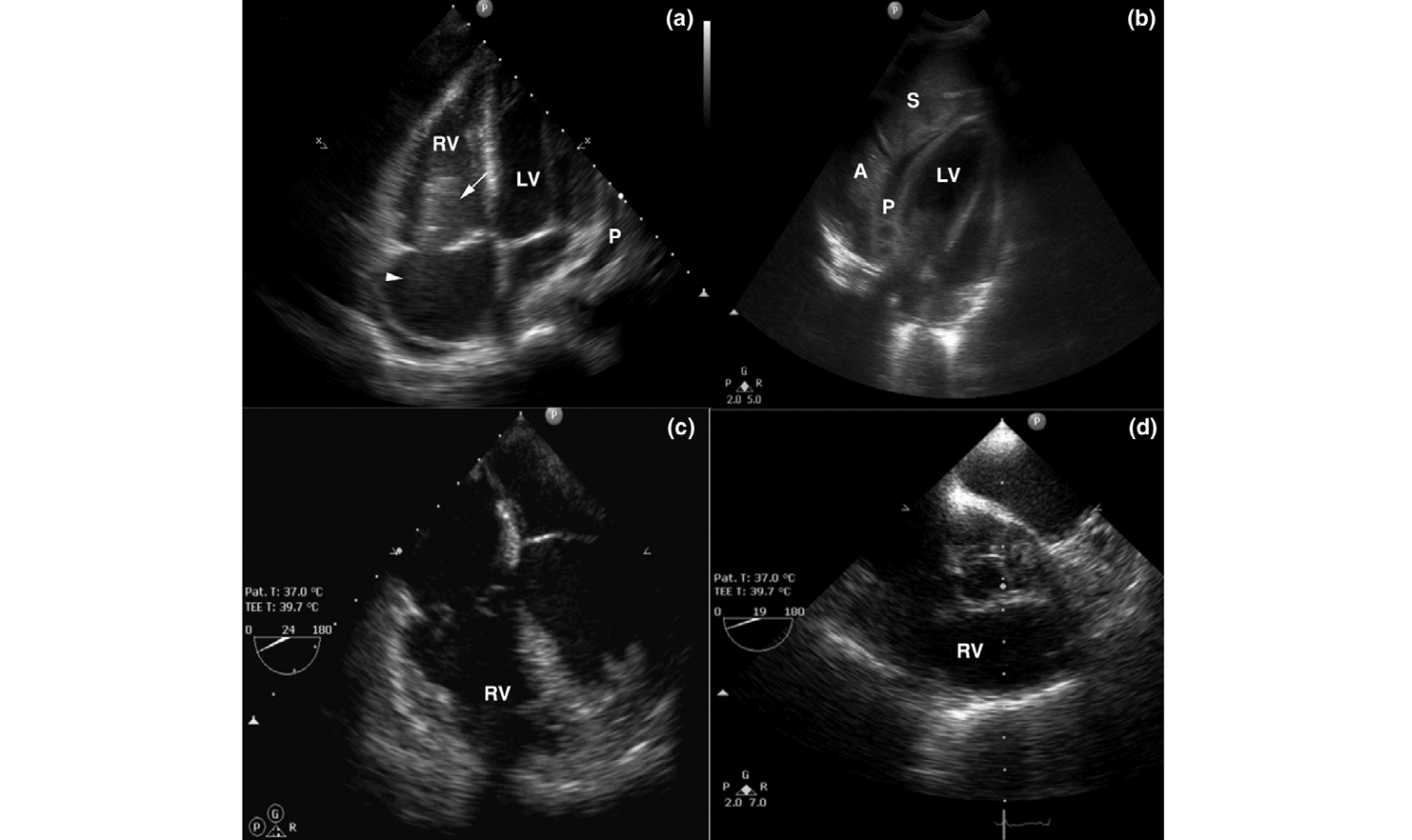
**Chest echography (a,b) and transesophageal echocardiography (c,d) (LV, left ventricle; P, pericardial fluid; S, spleen)**. **(a) **Apical four-chamber view of the artifact in the right ventricle; **(b) **echography demonstrating the lung atelectasis, the spleen and the pericardial fluid in the same patient; and **(c,d) **transesophageal echocardiography, revealing no abnormalities.

The cardiac artifact was observed in 17 out of 205 patients (8,29%) in whom signs of lung atelectasis and/or pleural effusion were evident on lung echography. Atelectatic lung associated with pleural effusion was mainly present in the lower parts of the anterior, lateral, and posterior chest wall. Furthermore, these echographic findings were correlated to chest radiography, which revealed signs of underlying lung pathology in the same regions of the lung. The configuration that exhibited the respiratory variation was observed in 10 patients (58.8%), whereas in seven patients (41.2%) the pattern of respiratory variation was not fully apparent. The artifact was observed within the left cardiac chambers in 11 cases (64.7%) and within the right cardiac chambers in six (35.3%; Figure 3). Consequently, transthoracic echocardiography was performed in all 17 patients, but it failed to visualize the 'mass' (Figure 3). Twelve out of 17 patients (70.5%) died, whereas in all five survivors the artifact resolved upon normalization of the lung echographic findings. There were no inter-observer variations in identification of the artifact, and no intra-observer variations were recorded (agreement 100% in all sequences). Hence, we termed this 'mass', presumably due to atelectatic or consolidated lung and/or pleural effusion, the 'cardiac-lung mass' artifact.

## Discussion

The utility of lung and cardiac echography is well established in the ICU setting. They both permit noninvasive, rapid, and reproducible evaluation of the respiratory and cardiac status of critically ill patients at the bedside [6,15,19,20]. The present study offers two simple messages. First, lung echographic data may add invaluable information to echocardiographic data. Hence, the general chest ultrasound examination is a powerful diagnostic and monitoring tool in the ICU. Second, areas of atelectasis and/or pleural effusion in the critical care patient may lead to the generation of echocardiographic artifacts with mobile components, which resemble the pattern of motion of pathologic lung regions observed by lung echography.

The findings presented here show that echocardiographic artifacts may present as intracardiac configurations resembling 'pseudomasses' that may be attributable to beam width artifact, generated by anomalies of the lung parenchyma. Consequently, these artifacts could not be depicted by TEE and resolved upon normalization of lung echographic findings [25,26]. The ultrasound beam exists in a three-dimensional volume, and bright reflectors recorded from different tomographic planes may be misinterpreted as being part of the image [27]. Additionally, apart from the main beam, ultrasound probes emit secondary beams (side lobes) propagating radially from the center of the main beam. A returning echo produced by a strong reflector located in the side lobes will be displayed as if it originated from the main beam [18-20,27]. Strong reflectors such as the pathologic lung parenchyma may have participated in the formation of the cardiac artifact. In the cases described here, the 'mass' could have been a mirror image artifact of atelectatic lung projecting into the heart. Structures such as atelectatic lung areas and/or areas of pleural effusion immediately adjacent to highly acoustic interfaces, such as the diaphragm, may appear to be duplicated because of the scattering of the sonic signal [28-31]. Hence, the recording of 'off-axis' information results in the formation of a double image of such structures, which may be misplaced, distorted, incompletely portrayed, or entirely 'off-axis' [28-31].

In our cases, neither intra- nor inter-observer variations were recorded for the identification of the artifact, but past studies clearly demonstrated that assessment of mobile echoes by transthoracic echocardiography is difficult [7]. There are no clear echocardiographic criteria and/or consensus for their identification [7]. The high reproducibility and inter-observer agreement in our cases was presumably due to the fact that the pathologic lung structures, responsible for the generation of the artifact, provided rather consistent imaging data, and hence a steady source of distortion of the ultrasound signal, during the study period. It is of note that the vast majority of the patients who exhibited the artifact subsequently died. However, the clinical importance of these findings remains to be confirmed by future studies. In the present series, a rather long period of hospitalization in the ICU was documented, corresponding to a longer duration of mechanical ventilation and therefore predisposing to increased incidence of pulmonary complications, but this was not an end-point of the study. Furthermore, this by no means indicates that similar ultrasound findings could not be observed in the acute critical care setting, because they correspond to underlying lung pathology that could occur at any time during ICU hospitalization. Finally, the present study was mainly observational and rather focused upon imaging findings and upon characterization of the artifact.

Despite their obvious utility, lung and cardiac ultrasound have significant limitations [26,32]. Knowledge of normal anatomical variants that can mimic pathological lesions, familiarity of the observer with basic ultrasound physics, and understanding of extracardiac echo patterns are indispensable for differentiating an artifact from a true cardiac anomaly [27-31]. The confirmation or refutation of mobile intracardiac artifact by TEE and a progressive evaluation of all imaging findings are vital steps in the final clinical assessment. Indeed, the role played by TEE is mandatory for excluding or confirming the presence of intracardiac masses, especially in mechanically ventilated patients. Multiple acoustic windows and views can be obtained by TEE, thus facilitating the differential diagnosis of intracardiac artifact from true cardiac anomalies. Assessment may be a difficult diagnostic dilemma for the clinician, especially in the ICU setting, in which the risk for thrombus formation, infection, and other complications is increased [7,24-27].

## Conclusion

Lung atelectasis, consolidation, and/or pleural effusion may create a mirror image, intracardiac artifact in mechanically ventilated patients. The latter was termed the 'cardiac-mass lung' artifact, to emphasize the important diagnostic role of both echocardiography and lung echography in these patients. Such mobile intracardiac artifacts are rare, but they may alert the clinician to search for possible signs of corresponding lung pathology.

## Key messages

• Lung atelectasis, consolidation and/or pleural effusion may create a mirror image, intracardiac artifact in mechanically ventilated patients, which we termed the 'cardiac-lung mass' artifact, to emphasize the important diagnostic role of both echocardiography and lung echography in these patients.

• The artifact resembles the form of an intracardiac 'mass', which is visible by transthoracic echocardiography, mainly on apical views. Furthermore, on M-mode it exhibits a pattern of respiratory variation similar to the lung 'sinusoid sign'.

• Despite the fact that the presence of the 'cardiac-lung mass' artifact occurs rarely (<10%) in patients with adjacent lung pathology, it may alert the clinician to search the affected lung areas thoroughly for pathophysiologic alterations.

## Abbreviations

APACHE: Acute Physiology and Chronic Health Evaluation; ICU: intensive care unit; TEE: transesophageal echocardiography.

## Competing interests

The authors declare that they have no competing interests. No financial support was received for this study.

## Authors' contributions

AK conceived of the study, participated in the design of the study, and drafted the manuscript. TS participated in the design of the study, performed both echographic methods in the ICU setting, and drafted the manuscript. DK participated in the design of the study, performed both echographic methods in the ICU, provided expert imaging and ultrasound analysis, and drafted the manuscript. DL performed both echographic methods in the ICU and provided expert advice on lung echographic findings. JP performed both echographic methods in the ICU and drafted the manuscript. CY participated in the design of the study, provided expert analysis upon ultrasound data, and drafted the manuscript. CS participated in the design of the study, provided expert echocardiographic consulting, and helped to draft the manuscript. All authors read and approved the final manuscript.
